# Theaflavin Induces Apoptosis of A375 Human Melanoma Cells and Inhibits Tumor Growth in Xenograft Zebrafishes Through P53- and JNK-Related Mechanism

**DOI:** 10.3389/fphar.2020.01317

**Published:** 2020-08-31

**Authors:** Lei Zhang, Bo Yan, Shijie Meng, Li Zhou, Yiqiao Xu, Wenxi Du, Letian Shan

**Affiliations:** ^1^School of Biological and Chemical Engineering, Zhejiang University of Science and Technology, Hangzhou, China; ^2^The First Affiliated Hospital, Zhejiang Chinese Medical University, Hangzhou, China; ^3^Research and Development Department, Hunter Biotechnology, Inc., Hangzhou, China

**Keywords:** green tea, theaflavin, melanoma, zebrafish, P53, JNK

## Abstract

Theaflavin (TF) is a major active pigment and polyphenol of tea, possessing anti-cancer activities. However, little is known about its activity and mechanism on melanoma cells. To fill this gap, we conducted *in vitro* experiments (cell viability assay, morphology observation, DAPI staining, and flow cytometry) and *in vivo* experiment by using a xenograft model of larval zebrafishes. Real-time PCR (qPCR) and Western blot (WB) analyses were conducted to explore the mechanism of TF. The *in vitro* data showed that TF exerted significant anti-proliferative and pro-apoptotic effects on A375 cells in a concentration-dependent manner. *In vivo*, TF significantly inhibited A375 tumor growth in larval zebrafishes at 0.67 and 2.0 μg/ml (1.3 to 3.9 μM). qPCR and WB data showed that TF significantly activated the P53 pathway-related proteins (ATM, CHK1/2, P53, and CASP8/3) and the JNK pathway-related proteins (ASK1, JNK, and C-JUN) through phosphorylation and cleavage, followed by activation of pro-apoptotic molecules (PARP, *BAX*, *BIM*, *PUMA*, and *P53*). In sum, TF possessed cytotoxic pro-apoptotic and tumor-inhibitory effects on A375 cells through activations of P53 and JNK pathways. This is the first report on TF regarding its effects and mechanism on A375 cells, making it a promising candidate of natural products for clinical treatment of melanoma.

## Introduction

Melanoma is a fatal type of skin cancer, with high metastatic potential and intractability (Yang et al., 2018). Melanoma patients at advanced stages are always unresectable and have a worse prognosis (Leonardi et al., 2018). Chemotherapy is a mainstay of clinical treatment for melanoma, but the efficacies of chemotherapeutics are limited due to their side effects on healthy tissues (Oliveira Pinho et al., 2019). Recently, several immunotherapies, including CTLA-4 or PD-1 receptor inhibitors, have been developed as new options for treating melanoma, but their efficacies are unreliable and their side effects are still non-negligible (Rodríguez-Cerdeira et al., 2017). Therefore, new strategies with satisfactory effectiveness and safety are urgently needed. Traditional Chinese medicine (TCM) is a complementary approach and has been clinically applied for thousands of years, possessing certain efficacy and fewer side effects. According to the TCM theory, cancer is associated with body accumulation of phlegm, toxins or inflammation, and the anti-phlegm, anti-toxic, or anti-inflammatory herbs may possess anti-cancer effects (Wang and Cheng, 2019). The theory has been supported by many cases. For example, curcumin in *Rhizoma Curcumae longae* exerted cytotoxic effects by inducing apoptosis and inhibiting angiogenesis of melanoma cells (Mirzaei et al., 2016), and vitexin in *Vitex negundo* suppressed melanoma cell growth by inducing DNA damage and increasing ROS levels (Liu et al., 2018). Therefore, TCM herbal components have great potential for treatment of cancers, such as melanoma.

Tea [*Camellia sinensis* (L.) O. Kuntze] is one of the most prevalent beverages in the world. It is well-known not only for the peculiar flavor but also for the benefits to health. Due to the difference of process, tea has three main types, including unfermented green tea, partially fermented *oolong* tea, and fully fermented black tea or *pu-erh* tea (Kuo et al., 2005). Tea leaves have been characterized as a TCM herb with anti-phlegm and anti-toxic properties, indicating its anti-cancer potential. Modern studies have reported that tea drink is effective in preventing and treating cancers (Jin et al., 2018). Recently, tea polyphenols have been found to possess anti-cancer activity, which have superior effect to tea drink (Mao et al., 2019). Theaflavin (TF) is such a polyphenol component produced by oxidation of catechins of tea leaves during fermentation, acting as a main pigment for the color, flavor and bioactivity of tea (Roberts et al., 1957). It is capable of inducing apoptosis in a variety of cancer cell lines, such as human breast carcinoma cell lines (MCF-7, MDA-MB-231, T47D, and ZR-75-1), colon carcinoma cell lines (HCT-15 and HT-29), and hepatic carcinoma cell lines (HCCLM3 and Huh-7), indicating anti-cancer potential (Adhikary et al., 2009; Lahiry et al., 2010; Li et al., 2012; Shao et al., 2016). Nevertheless, little attention has been given to its effect towards melanoma as yet.

In view of the reported pro-apoptotic effects of TF on many cancer cell lines (Lahiry et al., 2008), we put forward a hypothesis that TF exerts pro-apoptotic effects on melanoma cells. To verify this, the present performed *in vitro* experiments to evaluate the cytotoxic pro-apoptotic effect of TF on human melanoma cells and conducted *in vivo* experiment by using a xenograft model in larval zebrafishes to determine its tumor-inhibitory effect. Moreover, the mechanism of TF was also explored.

## Materials and Methods

### Materials and Chemicals

Theaflavin (TF, >95% of purity) was provided by Theabio Co., Ltd (Hangzhou, China) (Batch number: 20181211061). Dulbecco′s modified Eagle′s medium (DMEM) containing high glucose (4.5 g/l) was obtained from HyClone Laboratories (UT, USA). Fetal bovine serum (FBS) was obtained from Cell Max (Beijing, China). Trypsin (0.25%) were obtained from Gibco (NY, USA). 3-(4,5-dimethylthiazol-2-yl)-2,5-diphenyltetrazolium bromide (MTT) and dimethyl sulfoxide (DMSO) were obtained from Sigma (St. Louis, MO, USA). Annexin-V: FITC apoptosis detection kit was obtained from BD Biosciences (CA, USA). 4’-6-diamidino-2-phenylindole (DAPI) staining solution was obtained from Thermo Fisher Scientific (MA, USA). Primary antibodies were obtained from Cell Signaling Technology (MA, USA). Trizol reagent and real time polymerase chain reaction (real time PCR) kit were obtained from TaKaRa (Dalian, China).

### Cell Line Preparation

Human HFF-1 skin fibroblast and A375 melanoma cell line were obtained from Shanghai Cell Bank of Chinese Academy of Sciences (Shanghai, China), and human A875 melanoma cell line was obtained from Kunming Cell Bank of Chinese Academy of Sciences (Kunming, China). These cell lines were cultured in DMEM medium containing 10% FBS at 37°C in a humidified 5% CO_2_ incubator. The medium was daily changed, and the cells were treated with TF in their logarithmic growth phase.

### Zebrafish Preparation

Wild-type AB strain of zebrafishes was obtained from the China Zebrafish Resource Center, Institute of Hydrobiology, China Academy of Science (Wuhan, China) and accredited by the Association for Assessment and Accreditation of Laboratory Animal Care International (SYXK 2012-0171). Larval zebrafishes at 2 dpf (days post fertilization) were produced by natural pair-mating and housed in a light-controlled aquaculture facility with a standard 14:10 h day/night photoperiod and fed with live brine shrimp twice a day and fry flakes once a day.

### Cell Viability Assay and Morphological Observation

MTT assays were conducted to determine the inhibitory effects of TF on melanoma cell lines, as previously described (Zhou et al., 2017). Cells were seeded into 96-well plates at 6×10^3^ cells/well in 200 μl medium for 24h adherence, followed by treatment with TF at concentrations of 0, 50, 100, 150, 200, 250, 300, and 400 μg/ml for 24, 48, and 72 h. Then 20 μl of MTT solution (5.0 mg/ml) was added to each well and incubated at 37°C for 4h. DMSO (150 μl) was added in each well and the optical density value (OD value) was measured at 490 nm with Biorad microplate reader (CA, USA). Inhibitory rate (%) = [1-(TF-treated OD/untreated OD)] × 100%. The 50% inhibitory concentrations (IC_50_) for 24, 48, and 72 h were calculated by regression analysis. Accordingly, 120, 240, and 360 μg/ml (232.3, 464.7, and 697.0 μM) were designated as low, middle, and high doses of TF. Then, HFF-1, A375, and A875 cell lines were seeded into 96-well plates as above, and treated with middle dose of TF at 24 h. The cell morphology of A375 cells was observed under Carl Zeiss fluorescence microscope (Göttingen, Germany).

### Apoptosis Analysis by DAPI Staining and Flow Cytometry

Cell apoptosis was determined by DAPI staining and annexin-V/PI staining-based flow cytometry. For DAPI staining, A375 cells were seeded into 96-well plates and treated with TF at low, medium, and high concentrations for 24 h, followed by fixation with 4% paraformaldehyde in PBS for 30 min at room temperature and staining with DAPI for 10 min in dark. After thrice wash, cells were observed using five coverslips under Carl Zeiss fluorescence microscope (Göttingen, Germany) and the apoptotic cells were counted. Flow cytometry was conducted according to the manufacturer′s instruction. Briefly, A375 cells were seeded into 6-well plates at 3×10^5^ cells/well for 24 h and treated with TF at low, medium, and high concentrations for another 48 h. Afterwards, the cells were washed twice and labeled with annexin V-fluorescein isothiocyanate solution and PI in binding buffer. Fluorescence intensity of the cells was detected by BD C6 flow cytometry (CA, USA). The analysis was replicated and the early apoptotic and late apoptotic cell rates (%) were calculated.

### Xenograft Animal Assay

For determining the dose range of TF, totally 300 larval zebrafishes at 3 dpf were used and randomly cultured into 6-well plates with 30 fishes each. TF were dissolved into each well at 0, 3.47, 10.4, 31.25, 62.5, 125, 250, 500, 1,000, and 2,000 μg/ml, respectively, for 24 h. Afterwards, fishes in each group were observed under a stereoscopic microscope to record mortality and adverse events. As described by our previous study, no observed adverse effect level (NOAEL) of TF was estimated, and 1/9 NOAEL, 1/3 NOAEL and NOAEL were applied as the low, middle, and high doses for the following experiment (Jin et al., 2018).

To establish the xenograft model, A375 cells were stained with CM-Dil (red fluorescence) at a dilution of 1:1,000 and microinjected into the yolk sac of larval zebrafishes (2 dpf) at a dose of 200 cells/fish. After tumor growth for 24 h, all fishes were observed under a fluorescent microscope (AZ100, Nikon, Tokyo, Japan) for model verification. The A375-bearing fishes were grouped into 5 groups (30 fishes each) and treated with 0 μg/ml, 1/9 NOAEL, 1/3 NOAEL, and NOAEL of TF, as well as 15 μg/ml (50 μM) of cisplatin, respectively, for 24 h. The fluorescence intensity (FI) of A375 cell mass of zebrafishes was detected and the inhibitory rate was calculated as: inhibitory rate (%) = [1–(FI of treated group/FI of untreated group)] × 100%.

### Real Time PCR (qPCR) Analysis

To reveal the molecular actions of TF on A375 cells, qPCR was employed on an ABI QuantStudio™ 7 Flex Real-Time PCR System (Applied Biosystems, CA, USA). The total RNA of A375 cells was extracted using Trizol reagent and synthesized to cDNA *via* reverse transcription. The qPCR reaction system had a 20.0 μl volume: 10 μl SYBR^®^ Premix Ex Taq II (Tli RnaseH Plus), 0.8 μl PCR forward primer, 0.8 μl PCR reverse primer, 2.0 μl template cDNA, 0.4 μl ROX reference dye, and 6.0 μl ddH_2_O. The qPCR reaction condition was set to 95°C for 30 s initial denaturation, 40 cycles of 95°C for 5 s denaturation, 60°C for 34 s annealing, and 72°C for 40 s extension. At the end of each reaction, a melting curve analysis was performed. *β-ACTIN* was used as the reference gene and the 2^-ΔΔCT^ method was applied to analyze the relative expression of each gene (**Table 1**).

**Table 1:** Primer sequences used for qPCR analysis.

Gene	Forward primer	Reverse primer
*β-ACTIN*	5′-CATGTACGTTGCTATCCAGGC-3′	5′-CTCCTTAATGTCACGCACGAT-3′
*BAX*	5′-CCTTTTCTACTTTGCCAGCAAAC-3′	5′-GAGGCCGTCCCAACCAC-3′
*BCL-2*	5′-ATGTGTGTGGAGAGCGTCAACC-3′	5′-TGAGCAGAGTCTTCAGAGACAGCC-3′
*BIM*	5′-ACCAAACCAAAGCCGTCATCA-3′	5′-GGAGCCAGTAAACGTATTGGAAG-3′
*C-MYC*	5′-GCCACGTCTCCACACATCAG-3′	5′-TGGTGCATTTTCGGTTGTTG-3′
*P21*	5′-GGCAGACCAGCATGACAGATT-3′	5′-GCGGATTAGGGCTTCCTCT-3′
*P53*	5′-TCAACAAGATGTTTTGCCAACTG-3′	5′-ATGTGCTGTGACTGCTTGTAGATG-3′
*PUMA*	5′-GACCTCAACGCACAGTACGAG-3′	5′-AGGAGTCCCATGATGAGATTGT-3′

### Western Blot (WB) Analysis

The protein expression of A375 cells with TF treatment at 0 μg/ml and 120 μg/ml (232.3 μM) was analyzed by WB analysis. The total proteins were extracted using a lysis buffer (50 mM Tris-HCl pH 7.4, 150 mM NaCl, 1 mM EDTA, 1% Triton, 0.1% SDS, 5 μg/ml leupeptin, and 1 mM PMSF) for 30 min on ice with repeated freezing and thawing. Targeted proteins were separated using denaturing sodium dodecyl sulfate polyacrylamide gel electrophoresis (SDS-PAGE) (8~12%) and then transferred onto a polyvinylidene fluoride (PVDF) membrane (Millipore, MA, USA). The membrane was blocked with 5% non-fat milk for 2 h, followed by overnight incubation at 4°C with the antibodies against: ACTIN, ASK1, ATM, phosphorylated ATM (*p*-ATM), ATR, phosphorylated ATR (*p*-ATR), cleaved caspase 3 (*c*-CASP3), cleaved caspase 8 (*c*-CASP8), CHK1, CHK2, phosphorylated CHK1 and CHK2 (*p*-CHK1 and *p*-CHK2), JNK, phosphorylated JNK (*p*-JNK), C-JUN, phosphorylated C-JUN (*p*-C-JUN), cleaved PARP (poly ADP-ribose polymerase), P53, and phosphorylated P53 (*p*-P53). After incubation with the secondary antibody, these proteins were visualized with an enhanced chemiluminescence kit (Amersham Pharmacia Biotech, Little Chalfort, UK) and detected using a chemiluminescence analyzer.

### Statistical Analysis

Data were expressed as mean values ± SD and subjected to one-way ANOVA, followed by Fisher’s least significant difference (LSD) comparison. All analyses were performed using an updated version of DPS software (Tang and Zhang, 2013).

## Results

### Anti-Proliferative Effect of TF

**Figure 1:**
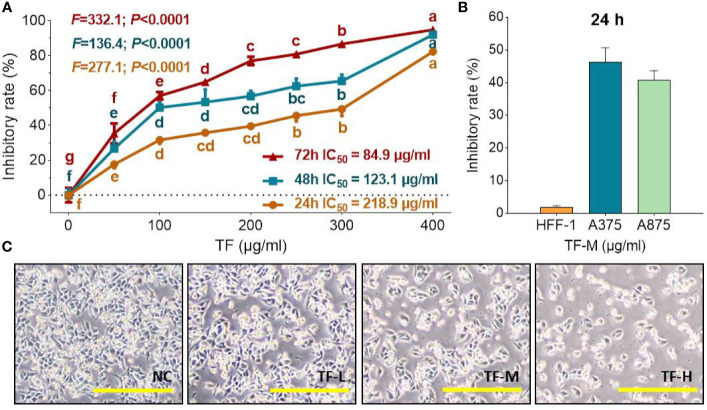
Cell viability of A375 cells with TF treatment at 24, 48, and 72 h **(A)**, cell viability of HFF-1, A375, and A875 cells with TF treatment at 24 h **(B)**, and morphology of A375 cells with theaflavin (TF) treatment at 24 h **(C)**. Data were mean ± SD (*n* = 5). By means of Fisher’s least significant difference (LSD) multiple comparisons, data (mean ± SD) with same lowercase letter (b vs. bc; bc vs. cd; c vs. c; cd vs. d; d vs. d) indicate no significant difference between each other, while data with different letters (a vs. b vs. c vs. b vs. e vs. f vs. g) indicate significant difference with each other. Scale bar = 200 μm.

As shown in **Figure 1A**, TF at 50 μg/ml (96.8 μM) significantly inhibited the viability of A375 cells, and the inhibitory rates were increased with increasing TF concentrations from 50 to 400 μg/ml (96.8 to 744.4 μM) (each *P* < 0.01 vs. normal level), indicating a concentration-dependent manner. The inhibitory rates were also increased with TF treatment from 24 to 72 h, with IC_50_ of 218.9 to 84.9 μg/ml (423.8 to 164.4 μM), respectively. Then, we applied 120, 240, and 360 μg/ml (232.3, 464.7, and 697.0 μM) as the doses of TF-L, TF-M and TF-H, respectively. As shown in **Figure 1B**, TF-M obviously inhibited the viability of A375 and A875 cells but exerted little effect on HFF-1 cells. As shown in **Figure 1C**, the morphology of A375 cells was obviously altered and the living cell number was decreased with TF treatment at increasing concentrations.

### Pro-Apoptotic Effect of TF

**Figure 2:**
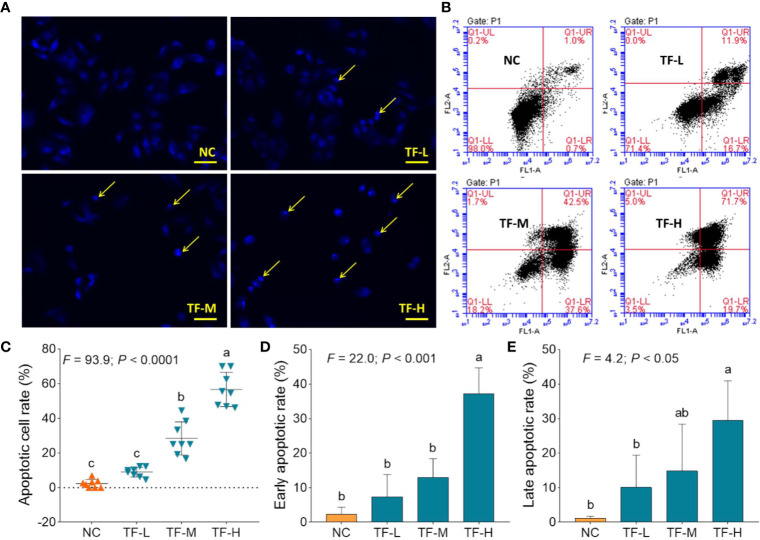
DAPI staining observation **(A)** and flow cytometry analysis **(B)** on A375 cells with theaflavin (TF) treatment. Statistical analysis of apoptotic cell rate **(C)**, statistical analysis of early apoptotic rate **(D)** and statistical analysis of late apoptotic rate **(E)**. By means of Fisher’s least significant difference (LSD) multiple comparisons, data (mean ± SD) with same lowercase letter (a vs. ab; ab vs. b; b vs. b; c vs. c) indicate no significant difference between each other, while data with different letters (a vs. b vs. c) indicate significant difference with each other. Scale bar = 100 μm.

DAPI staining and flow cytometry were performed to evaluate the pro-apoptotic effect of TF on A375 cells. The result of DAPI staining showed apoptotic morphology, including shrunken shape, karyopyknosis, and nuclear fragmentation, in A375 cells with TF treatment from 120 to 360 μg/ml (232.3 to 697.0 μM) (**Figure 2A**). The apoptotic cell numbers were significantly increased with TF treatment at 240 and 360 μg/ml (464.7 and 697.0 μM) (each *P* < 0.01 vs. NC) (**Figure 2C**). The result of flow cytometry showed TB-induced early apoptosis and late apoptosis of A375 cells (**Figure 2B**). The numbers of early and late apoptotic cells were increased with TF treatment from 120 to 360 μg/ml (232.3 to 697.0 μM) (**Figure 2B**), and their proportions were significantly higher with TF treatment at 360 μg/ml (697.0 μM) (*P* < 0.01 and *P* < 0.05 vs. normal level) (**Figures 2D, E**). The results indicated that TF induced apoptosis of A375 cells in a concentration-dependent manner.

### In Vivo Effect of TF on Xenograft Zebrafishes

**Figure 3:**
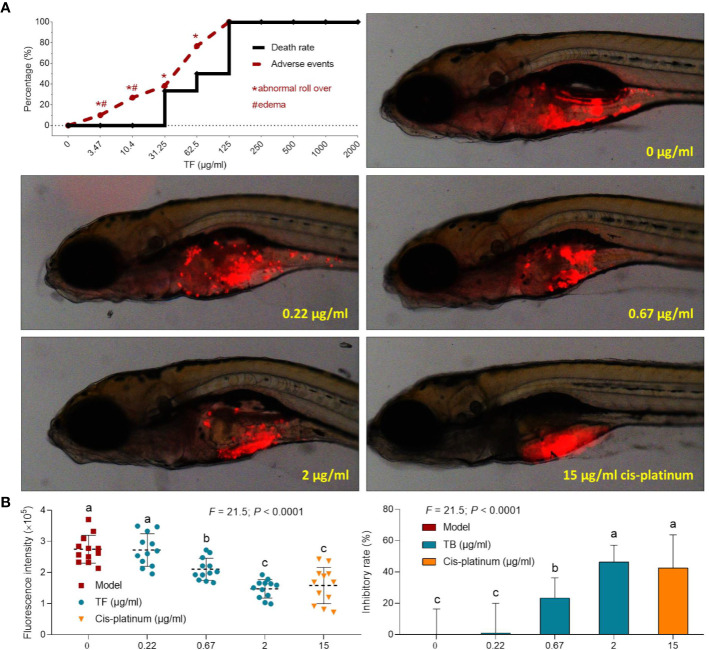
Mortality and adverse events of larval zebrafishes induced by theaflavin (TF) and observation of larval zebrafishes xenotransplanted with A375 cells with treatment of TF or cisplatin **(A)** as well as the fluorescence intensity and inhibitory rates of TF **(B)**. Fluorescent area in red represents the A375 cell mass. By means of LSD multiple comparisons, data (mean ± SD) with same lowercase letter (a vs. a; c vs. c) indicate no significant difference between each other, while data with different letters (a vs. b vs. c) indicate significant difference with each other.

The curves of mortality and adverse events of zebrafishes with TF treatment were shown in Fig. 3A. Fish death was caused by TF at 31.25 μg/ml (60.5 μM), and no fish was survived with TF at 125 μg/ml (242.0 μM), indicating the maximum non-lethal dose of TF less than 31.25 μg/ml (60.5 μM). The adverse events, including abnormal body roll over and edema, were observed with TF treatment from 3.47 μg/ml (6.7 μM) to its higher doses, indicating the NOAEL of TF less than 3.47 μg/ml (6.7 μM). After replicated tests, the NOAEL was finally estimated as 2.0 μg/ml (3.9 μM). Thereby, 0.22, 0.67, and 2.0 μg/ml (0.4, 1.3, and 3.9 μM) were used as the low, middle, and high doses of TF for the subsequent experiment.

As shown in **Figure 3B**, a xenograft model of A375 cells was established in larval zebrafishes and the fluorescent intensities of the cell mass in the fishes were tested. After 24 h treatment, TF from 0.22 to 2.0 μg/ml (0.4, 1.3, and 3.9 μM) obviously inhibited the A375 tumor growth, with inhibitory rates from 1.0 to 46.4%. The inhibitory effects of TF at 0.67 and 2.0 μg/ml (1.3 and 3.9 μM) were significant, if compared with the model group (*P* < 0.01), and the effect of TF at 2.0 μg/ml (3.9 μM) was even higher than that of cisplatin at its NOAEL (50 μM).

### Molecular Action of TF on mRNA Expressions in A375 Cells

**Figure 4:**
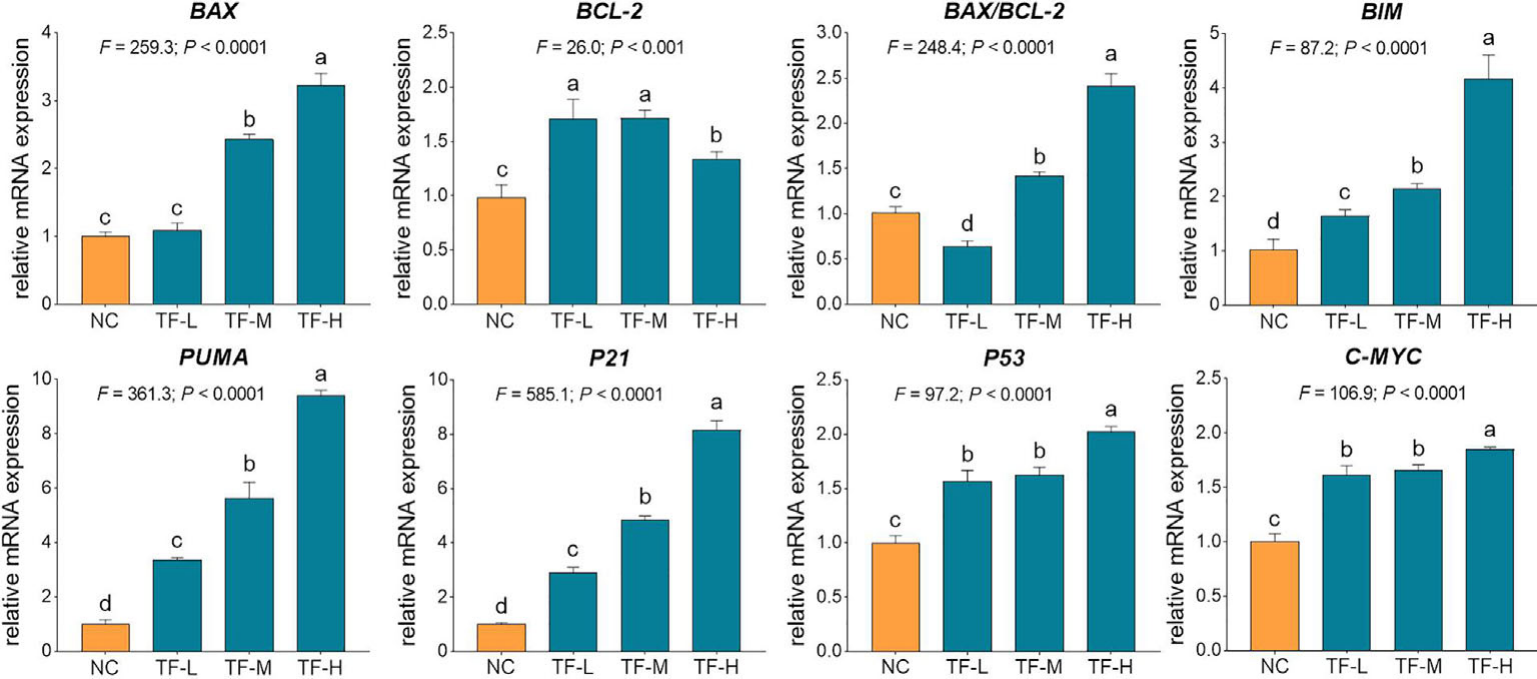
Relative mRNA expression of theaflavin (TF)-targeted genes in A375 cells after 24 h treatment. By means of Fisher’s least significant difference (LSD) multiple comparisons, data (mean ± SD) with same lowercase letter (a vs. a; b vs. b; c vs. c) indicate no significant difference between each other, while data with different letters (a vs. b vs. c vs. d) indicate significant difference with each other.

The relative mRNA expressions of TF-targeted genes were tested by qPCR assay. As shown in Fig. 4, the expressions of *BAX*, *BIM*, *C-MYC*, *P21*, *P53*, and *PUMA* were significantly up-regulated by TF (each *P* < 0.01 vs. NC level), except for that of *BAX* with TF treatment at its low concentration. Although the expression of *BCL-2* was up-regulated by TF, the ratios of *BAX/BCL-2* were significantly higher with TF treatment at its middle to high concentrations than that of NC level (*P* < 0.01), indicating the major role of *BAX* in the action of TF.

### Molecular Action of TB on Protein Expressions in A375 Cells

**Figure 5:**
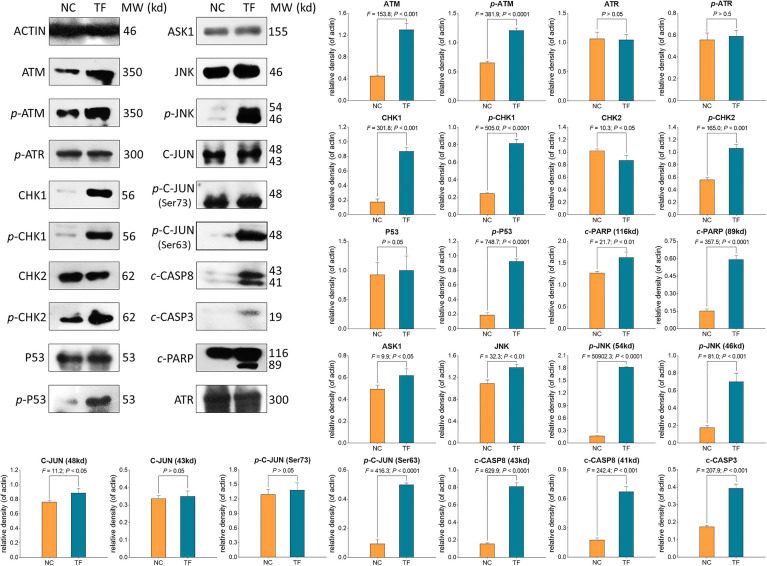
Expression and phosphorylation of theaflavin (TF)-targeted proteins in A375 cells after 24 h treatment. Data (mean ± SD) with different lowercase letters are significantly different with each other at Fisher’s least significant difference (LSD) multiple comparisons.

WB was applied to determine the expression and phosphorylation of proteins targeted by TF. As shown in **Figure 5**, the expressions of ATM, *p*-ATM, CHK1, *p*-CHK1, *p*-CHK2, *p*-P53, *c*-PARP, ASK1, JNK, *p*-JNK, C-JUN (48 kd), *p*-C-JUN (Ser 63), *c*-CASP8, and *c*-CASP3 were significantly up-regulated by TF at 120 μg/ml (232.3 μM) (each *P* < 0.01 vs. NC level). Besides, the actions of TF on ATR, *p*-ATR, CHK2, P53, C-JUN (43 kd), and *p*-C-JUN (Ser 73) were insignificant (each *P* > 0.05 vs. NC level).

## Discussion

Although the anti-cancer activities of TF has been well documented (Sur and Panda, 2017; Takemoto and Takemoto, 2018; Sajadimajd et al., 2020), the knowledge of its effectiveness on melanoma is still little. To fill this gap, the present study conducted *in vitro* and *in vivo* experiments to study the effects and mechanism of TF against melanoma cells. For the first time, we demonstrated the cytotoxic pro-apoptotic and tumor-inhibitory effects of TF on melanoma A375 cells. Its mechanism was suggested to be associated with P53 and JNK pathways. The innovation of this study is the finding of TF′s anti-melanoma efficacy, while previous reports only focused on TF′s effects on other tumors (Lin, 2002; Sur and Panda, 2017). Furthermore, this is also the first report on the molecular action of TF on JNK pathway, while the P53 pathway-associated mechanism of TF′s pro-apoptotic effect on carcinoma cells has been previously reported (Lahiry et al., 2008).

Recently, xenograft tumor models using larval zebrafishes have attracted increasing attention for anti-cancer studies, owing to the advantages of larval zebrafishes compared to other animal models: (1) the lack of immune rejection against human cells provides higher success rate for xenotransplantation; (2) body transparency provides *in vivo* visible observation of tumor growth and drug toxicity; and (3) large-scale generation and rapid organogenesis provides shorter experimental periods (Langheinrich, 2003; Pardo-Martin et al., 2010; Konantz et al., 2012). In this study, TF exerted dose-dependent inhibitory effect on A375 tumor mass in larval zebrafishes (**Figure 3B**), with inhibitory rate of 46.4% at its NOAEL (3.9 μM). The inhibitory rate was higher than that of cisplatin, indicating that TF was more effective than cisplatin within their respective safe dose range. Moreover, the inhibitory rate was higher than that of another tea pigment (theabrownin) (Jin et al., 2018), suggesting TF as the most effective component of tea. However, the effective *in vivo* dose range (1.3 to 3.9 μM) of TF is much lower than its effective *in vitro* dose range (96.8 to 774.4 μM). The reason for such difference may be that, after oral administration, the metabolized TF derivatives have higher effect than that of TF. It indicates that oral application may be more efficient than other routes for TF.

According to the dose conversion rule, the effective doses (1.3 and 3.9 μM) of TF in larval zebrafishes can be estimated as 0.03 to 0.09 mg/kg in human (Zhang et al., 2003). It suggests that oral administration of TF at such a low dose range may be effective in treating patients with melanoma, indicating a good cost-effectiveness of this compound. Up to our knowledge, there are only a few reports regarding the clinical application of TF. A double-blind, randomized, placebo-controlled, parallel-group trial has applied TF-enriched green tea extract to treat patients with mild to moderate hypercholesterolemia for 12 weeks (Maron et al., 2003). In that trial, the daily intake of TF was 75 mg in green tea extract, which significantly reduced total cholesterol, LDL-C, and triglyceride in hypercholesterolemic adults without observation of significant adverse events (Maron et al., 2003). In this study, the effective dose range of TF was much lower than the reported one, suggesting a greater potential of TF for melanoma treatment in clinic. However, although the effective dose range of TF was small, its lethal dose threshold to zebrafish larvae was also low (<60.5 μM) and was lower than other tea pigments (**Figure 3A**) (Jin et al., 2018), leaving a concern for the potential toxicity of TF to human beings. Interestingly, we found that TF exerted little effect on the normal cell line (HFF-1 skin fibroblast) (**Figure 1B**), suggesting a clinical feasibility for its external use which can avoid the potential toxicity of its internal use.

Our mechanistic experiment showed that TF activated ATM, CHK1/2, P53, CASP8/3 in P53 pathway and also activated ASK1, JNK, and C-JUN in JNK pathway, associating with A375 cell apoptosis. The apoptosis was determined by DAPI staining and flow cytometric analysis at cellular level (**Figure 2**) and mediated by the overexpression of pro-apoptotic genes (*P53*, *BAX*, *BIM*, and *PUMA*) and the activation of apoptosis-related proteins (caspases and PARP) at the molecular level (**Figures 4** and **5**). *P53* (*TP53*) encodes a DNA-binding nuclear phosphoprotein with tumor suppressor activity, which acts as transcription factor at the center of a network for the control of apoptosis in response to cellular stresses (Martin et al., 2002). It activates apoptosis by stimulating the transcription of Bcl-2 family genes, such as *BAX*, *BIM*, and *PUM*A (Levine and Oren, 2009). *BAX* and *BIM* encode pro-apoptotic members that provoke apoptosis and cell death by activating caspase cascade in response to apoptotic stimuli (Rossé et al., 1998; Youle and Strasser, 2008). *PUMA*, as a *P53* up-regulated modulator of apoptosis, encodes a BH3 domain-containing protein that localizes to the mitochondria, interacts with Bax and Bak, and activates the caspase cascade by cleavage of CASP3 (Nakano and Vousden, 2001; Letai, 2009). In this study, CASP8 and CASP3 were cleaved in response to P53 activation. CASP8 is an initiating caspase in the apoptotic cascade, which activates CASP3 for apoptotic DNA fragmentation, resulting in subsequent cleavage of PARP (*c*-PARP) to execute the apoptotic process (Jänicke et al., 1998; Stennicke et al., 1998; Boulares et al., 1999). PARP is responsible for DNA repair and cell viability in response to exogenous stress (Satoh and Lindahl, 1992). It can be cleaved by CASP3 and thereby facilitates the cellular disassembly in apoptosis (Oliver et al., 1998). Thus, the amount of cleaved PARP (*c*-PARP) can be used as marker of cell apoptosis.

In our previous studies, we have reported that DNA damage induction was associated with P53 pathway-mediated pro-apoptotic mechanism of theabrownin (Wu et al., 2016; Jin et al., 2018). However, although both TF and theabrownin activated P53 pathway and induced tumor cell apoptosis, we did not find DNA damage induction with TF treatment in this study. Alternatively, we found the activation of ASK1–JNK–C-JUN cascade, which also functions as apoptotic pathway. In this signaling module, ASK1 (apoptosis signal-regulating kinase 1) is a mitogen-activated protein kinase that plays a key role in cytokine- and stress-induced apoptosis by triggering mitochondria-dependent pathway (Matsuzawa and Ichijo, 2001; Zhang et al., 2003). It activates downstream JNK signaling in response to different types of stress, leading to cell apoptosis through C-JUN activation and subsequent overexpression of pro-apoptotic genes (Tobiume et al., 2001). Chemotherapeutics, such as cisplatin, docetaxel, and paclitaxel, have been reported to induce apoptosis of melanoma cells through the JNK pathway independent of the P53 pathway (Mandic et al., 2001; Mhaidat et al., 2008; Selimovic et al., 2008). This indicates that TF might have an advantage compared to these drugs due to its dual-pathway-mediated mechanism of action, which has been preliminarily demonstrated by the higher tumor-inhibitory effects of TF than that of cisplatin in this study. However, there are some limitations of this study as follows: (1) the actions of P53 and JNK pathways in the proposed dual-pathway-mediated mechanism of TF has not been verified; (2) the interaction between P53 and JNK pathways has not been investigated, and which pathway plays the main role is unknown; and (3) the *in vitro* dose range and the *in vivo* dose range are quite different, the reason of which has been explained but needs experimental evidence. To address these issues, further studies are needed in future. For instance, siRNAs or inhibitors of P53 and JNK should be used to verify the actions of these pathways and to explore the interaction between each other, and the serum metabolites of TF should be chemically analyzed and pharmacologically studied to test our hypothesis that the metabolized TF derivatives have higher effect than that of TF. Moreover, since there are no clinical reports of TF for cancer treatment, the therapeutic efficacy and benefits of TF on cancer patients should be further studied.

## Conclusion

Since the anti-cancer potential of TF has been well documented, it remains uncertain whether TF is effective in treating melanoma. In this study, by using melanoma cell line and xenograft zebrafish model, we found cytotoxic pro-apoptotic and tumor-inhibitory effects of TF on melanoma cells and revealed its mechanism in association with the activations of P53 and JNK pathways. This is the first study describing the effects and mechanism of TF against melanoma cells. Since the mechanism of TF was not only dependent on the P53 pathway, it can be expected that TF may be effective in treating P53-mutated cell lines. Further studies are warranted to verify this deduction. Altogether, this study provides evidence for the efficacy of TF against melanoma, which contributes to the development of TF-derived agents for melanoma therapy.

## Data Availability Statement

The raw data supporting the conclusions of this article are available from the corresponding author on reasonable request.

## Ethics Statement

The animal study was reviewed and approved by the Ethics Committee of Zhejiang Chinese Medical University.

## Author Contributions

LeZ performed the main work of this paper. BY conducted the cellular and molecular experiments. LiZ and SM contributed to the writing of this manuscript. WD provided ideas and funding support to this work. YX conducted the zebrafish experiment. LS designed this work and drafted the manuscript. TE improved the design and draft of this paper. All authors contributed to the article and approved the submitted version.

## Funding

This work was supported by the National Natural Science Foundation of China (Grant No. 81774331, 81873049, and 81973873), the Zhejiang Provincial Natural Science Foundation of China (Grant No. LY18H270004), the Zhejiang Provincial Science and Technology Project of Traditional Chinese Medicine of China (Grant No. 2016ZZ011), the Zhejiang Provincial Key Construction University Superiority Characteristic Discipline (Traditional Chinese Pharmacology), and the Opening Foundation of China (Grant No. ZYX2018006).

## Conflict of Interest

YX was employed by the company Hunter Biotechnology, Inc.

The remaining authors declare that the research was conducted in the absence of any commercial or financial relationships that could be construed as a potential conflict of interest.

## Abbreviations

DAPI, 4’-6-diamidino-2-phenylindole; DMEM, Dulbecco′s modified Eagle′s medium; DMSO, dimethyl sulfoxide; dpf, days post fertilization; FBS, fetal bovine serum; FI, fluorescence intensity; LSD, Fisher’s least significant difference; NOAEL, no observed adverse effect level; MTT, 3-(4,5-dimethylthiazol-2-yl)-2,5-diphenyltetrazolium bromide; PARP, poly ADP-ribose polymerase; SDS-PAGE, denaturing sodium dodecyl sulfate polyacrylamide gel electrophoresis; TCM, theaflavin; TF, Traditional Chinese medicine.
